# Being here First: Ethnic Majority Children’s Autochthony Beliefs and Attitudes toward Immigrants

**DOI:** 10.1007/s10964-019-01015-0

**Published:** 2019-04-06

**Authors:** Maykel Verkuyten, Jochem Thijs

**Affiliations:** Ercomer, Faculty of Social and Behavioral Sciences, Utrecht University, Padualaan 14, 3584 CH Utrecht, P.O. Box 80.140, Utrecht, TC 3508 The Netherlands

**Keywords:** Autochthony, Collective Ownership, Attitudes, Immigrants, Early Adolescents

## Abstract

Autochthony belief (“that a country is owned by its first inhabitants”) can be an acceptable reason for claiming collective ownership of a territory and this claim can have negative consequences for newcomers. Children might reason that a place belongs to their in-group because “we” were here first and therefore have negative out-group attitudes. In three studies among Dutch majority group children (*N* = 879; *M*_*age*_ = 10.13 to 10.84, *SD* = 0.82 to 0.98; 49.7 to 54.5% girls), the expected negative association between autochthony beliefs and attitudes was found for different measures of ethnic attitudes, and was robust across gender, age, immigrant target group, ethnic identification, perceived multicultural education and classroom composition. Additionally, the association was especially strong among ethnic majority children who felt less at home in their own country but at the same time cared about being Dutch. It is concluded that a focus on autochthony belief makes a novel and relevant contribution to the intergroup developmental literature and to our limited understanding of children’s attitudes toward immigrant groups and newcomers more generally.

## Introduction

Almost all politicians in Western Europe today – from across the political spectrum – apparently believe that some people are more entitled to inhabit particular places than others. Their belief is usually based on a form of ‘primordial reasoning’ [‘we were here first’], where places are owned by ‘native’ groups who enjoy specific rights (prominent among them the right to feel at home) (Duyvendak 2011, p. 2).

Research on children’s ethnic, racial and anti-immigrant attitudes has focused on various factors and processes such as social-cognitive development, moral reasoning, in-group norms, group identification, intergroup contact, feelings of threat, and school composition and educational practices (see Levy and Killen 2008; Rutland and Killen 2015). What has received much less attention is the role of so-called lay theories for children’s negative out-group attitudes. There is some work on the ways in which group essentialism beliefs (Diesendruck and Menahem 2015), beliefs about the malleability or fixedness of human attributes (Levy and Karafantis 2008), protestant work ethic beliefs (Levy et al. 2008), and shared conflict beliefs (Bar-Tal and Teichman 2005) justify children’s negative attitudes. In addition to these lay theories, and similar to what is expressed in the quote above, children might reason that a place belongs to their in-group because they were there first and therefore that it is acceptable to exclude newcomers. In intergroup research, and following anthropological literature, this notion of primo-occupancy with the related ownership feelings has been labeled “autochthony beliefs” and these beliefs have been found to be important for adults’ ethnic out-group attitudes (e.g., Martinovic and Verkuyten 2013; Smeekes et al. 2015).

With three empirical studies conducted in the Netherlands the current research examines the importance of autochthony beliefs for majority group children’s (grades 4–6) attitudes towards immigrants. The current aim is to introduce the novel construct of autochthony to the intergroup development literature (Rutland et al. 2010) and to empirically examine its relevance for out-group attitudes of ethnic majority group children. The importance of this lay theory was investigated while considering ethnic identification, and by taking perceived multicultural education (Studies 2 and 3), classroom composition (Studies 2 and 3), and feeling at home in the country (Study 3) into account. The general expectation that was tested is that, whereas ethnic identification is mainly relevant for in-group attitudes, autochthony beliefs with their sense of native collective ownership (“it is ours”) are especially important for attitudes towards immigrant groups. More specifically, autochthony beliefs are considered to provide a justification for negative immigrant attitudes of majority group children, in particular when they at the same time do not feel at home anymore in their own country (see quote above). Thus, it was hypothesized that autochthony beliefs can serve to justify anti-immigrant attitudes and prevailing social inequalities (Jost et al. 2004). In short, this research breaks new ground in examining the importance of autochthony beliefs for children’s evaluation of immigrant groups and in investigating when these beliefs are particularly important by considering the moderating role of a sense of feeling at home in one’s country. In doing so we not only introduce the novel concept of autochthony to the literature but also make a contribution to the rather limited understanding of children’s attitudes toward the increasingly important group of immigrants (e.g., Brown 2011; Brown and Lee 2015).

Although young children (5 years) show tendencies to justify group advantages (Baron and Banaji 2009), the understanding and endorsement of lay theories that justify advantages appears later (Henry and Saul 2006; Levy and Karafantis 2008). It is only at around 8 years of age that children are able to use and weight different forms of information to assess and evaluate claims and rights (Smetana 2006). Furthermore, compared to objects, ownership of a territory might be a rather abstract issue for young children. Research has shown that children’s knowledge and beliefs about countries as geographical territories develops from around seven years onwards (see Barrett 2007). In addition, longitudinal research in Western Europe has shown that early adolescence is a sensitive developmental period for the emergence of negative attitudes toward immigrants (Gniewosz and Noack 2015). Therefore, the current research focuses on late childhood (grades 4–6) and there were no reasons to expect meaningful age differences. Lay theories once learned instigate a distinct and stable pattern of evaluation and judgment with respect to the target group. For example, research on protestant ethic beliefs and on entity and incremental beliefs indicates that these beliefs tend to function the same in social judgments regardless of age (Levy and Karafantis 2008).

### Ownership and Autochthony Beliefs

Perceived ownership is a pervasive notion that has profound implications for how individuals, think, feel and behave (Ye and Gawronski 2016). Ownership helps to organize the social and physical environment, regulates social interactions, and involves normative and moral privileges and responsibilities (Verkuyten and Martinovic 2017). Perceived ownership implies a bundle of rights such as the right to use what is owned and to decide whether to keep the target of ownership or not. Importantly, ownership also implies a ‘gatekeeper right’: the right to exclude others and to decide whether others are permitted or prohibited to use the object or have access to it (Merrill 1998). Ownership tells us not only what one may properly do to or with an object but especially what others may not do.

Considering these implications it is understandable that disputes over ownership of objects and places are among the most frequent and most intense intergroup conflicts (Toft 2014), also among children. Although ownership is not an obvious property of objects but rather abstract and imperceptible, young children already recognize it. Preschoolers have a basic understanding of ownership of physical objects and appreciate that owners are entitled to greater control over their property than non-owners (Kim and Kalish 2009; Rossano et al. 2011). By the age of 6 or 7 children’s notions of ownership are also applied to ideas and intellectual property (Shaw et al. 2012), and to places (O’Neal et al. 1977). There are many situations in which groups of children make claims on a particular physical place, such as when children convert a site in their play area, club or hideaway (Factor 2004). Territorial behavior whereby an intruder is excluded or punished for invading ‘our’ play area has been found in observational and experimental research among children (O’Neal et al. 1977; Zebian and Rochat 2012).

Children can infer ownership from seeing someone in possession of an object (Blake and Harris 2009), from verbal statements about who owns an object (Blake et al. 2012), from observing who decides on whether others can use it (Neary et al. 2009), and by using principles of past investment (creating or modifying an object), and ownership transfer (buying or giving) (Kanngiesser et al. 2014). In addition, children have been found to judge that an object belongs to the first person possessing it (Blake and Harris 2009; Friedman and Neary 2008). Older children and adults argue that the first person seen to possess a previously non-owned object is its owner (Friedman 2008; Friedman and Neary 2008), and the same has been found for the ownership of ideas (Shaw and Olson 2002). Similarly, being first at a particular place is information that children use to infer ownership. First arrival indicates one’s presence at a place before anyone else and this in itself is an important basis for establishing ownership. Experimental research has demonstrated that children as old as eight years infer personal (“mine”) and, importantly, also collective (“ours”) territorial ownership from first arrival (Verkuyten et al. 2015; Verkuyten et al. 2015). In this research, first arrival on an island was found to be a consideration that undermines the notion of equal sharing which is a key moral principle for children (e.g., Fehr et al. 2008; Rochat et al. 2009) that is typically applied when there are no other considerations involved, such as social conventions, group norms, interests and personal benefits.

These findings correspond with anthropological work on autochthony (Geschiere 2009) and “Sons of Soil” conflicts (Côté and Mitchell 2017) which demonstrates that primo-occupants are considered as rightfully possessing an area. This is evident in the moral and legal claims on resources and territory made by indigenous groups and so-called “first nations” or “first peoples”. The notion of autochthony suggest that first arrival determines place ownership with the related right to usage and to exclude others. The term autochthony can be traced back to ancient Greece and it means literally being “born from the soil” (Geschiere 2009). It is the belief that a country or a region belongs to its original inhabitants. This belief triggers self-evident notions of ownership and entitlements and thereby has an “implicit call for excluding strangers (‘allochthons’), whoever they may be” (Ceuppens and Geschiere 2005, p. 386). In European non-settler countries, this notion has been used to reject immigrants and to justify prejudice towards immigrant groups (Ceuppens 2011; Geschiere 2009). An immigrant represents someone trying to become a member of one’s national in-group and this typically elicits considerations of collective ownership and territorial belonging (Verkuyten and Martinovic 2017).

Autochthony can function as a justifying belief (Jost et al. 2004) because it makes the more advantaged position of the native majority group understandable and just. Children as young as 5 appear to be sensitive to these sorts of justifying beliefs (Baron and Banaji 2009) and 10-year olds have been found to endorse them (Henry and Saul 2006; Levy and Karafantis 2008). Furthermore, lay theories can justify children’s thoughts, feelings and behaviors toward minority out-groups (Levy et al. 2008). It was expected that majority group children who more strongly endorse autochthony beliefs will have a more negative attitude toward immigrant groups (Studies 1 to 3) and refugees (Study 2).

Furthermore, in Study 3 the expectation was tested that the association between autochthony beliefs and out-group attitudes is especially strong among children who feel less at home in the Netherlands (Barrett 2007; Verkuyten et al. 2014). Social identity development theory (Nesdale 2008) proposes that negative out-group attitudes tend to emerge when majority members feel that their position or well-being is undermined in some way by members of ethnic out-groups (e.g., Nesdale et al. 2005; Nesdale et al. 2005). Children’s attitudes and reasoning are influenced by social context and exposure (Killen et al. 2008) and research has shown that older children are aware of the societal debate on immigration and existing anti-immigration sentiments (Brown 2011). The Dutch immigration debate is typically framed in terms of what immigration means for the country, and the widespread sentiment is that the ethnic majority Dutch (“*Nederlander*” in Dutch language) feel estranged and no longer at home in their own country (“*Nederland*”): “The native Dutch, it is argued, have become like foreigners in their own country, feeling what foreigners should allegedly feel: not at home” (Duyvendak 2011 p. 98). Research shows that the ethnic Dutch do indeed increasingly (2006 to 2015) feel not at home in the Netherlands, and not feeling at home is higher among adolescents than adults (Huijnk and Andriessen 2016). This feeling can be expected to make autochthony beliefs more important for attitudes toward immigrant groups. Proprietary claims to a country that are accompanied by a sense of estrangement has been found to be associated with more negative ethnic out-group attitudes (Martinovic and Verkuyten 2013). Theoretically, and as indicated in the quote heading this paper, it is the combination of autochthony beliefs with the sense that one’s ability to feel at home in one’s own country is undermined, that in particular should be associated with more negative attitudes toward immigrant groups (Bobo 1999). Therefore, Study 3 tested the expectation that autochthony is stronger related to anti-immigrant attitudes among ethnic majority Dutch children who have a lower sense of feeling at home in the Netherlands.

### Ethnic Identification

Autochthony and the related sense of ownership does not have to imply a sense of ethnic group belonging. Individuals can believe that their group owns a particular place because of first-arrival without having a sense of commitment and belonging to in-group members (Verkuyten and Martinovic 2017). Research among children has demonstrated that stronger in-group identification goes together with more positive attitudes toward the in-group, including the national in-group (Bennett et al. 1998; Pfeifer et al. 2007). A positive evaluation of the group to which one belongs provides a positive sense of self (Tajfel and Turner 1979). This means that it can be expect that in the three studies majority group children with higher ethnic identification will be more positive about their ethnic majority group.

Higher in-group identification does not have to imply, however, that out-groups are evaluated more negatively (Cameron et al. 2001). Social identity development theory (Nesdale 2008) proposes that stronger feelings of in-group belonging lead to a stronger in-group orientation and commitment, but not necessarily to a rejection of out-groups. Higher compared to lower ethnic identifiers are predominantly focused upon and concerned about their ethnic in-group. Yet when concerns about the out-group come into play it is more likely that in-group identification is associated with negative out-group attitudes. According to social identity theory (Tajfel and Turner 1979), higher compared to lower identifiers are more sensitive to anything that could harm or undermine their feeling of in-group belonging. This means that it can be expected that the combination of autochthony beliefs with a sense of not feeling at home is particularly important for higher identifiers. Those who feel that the Netherlands is no longer their home and also consider being Dutch important to their sense of self are likely to base their negative attitudes toward immigrants on autochthony beliefs. This expected three-way interaction between autochthony, home feeling and ethnic identification was tested in Study 3.

### Current Study

The general expectation tested is that stronger autochthony belief is associated with more negative immigrant attitudes and that this association is particularly strong for children who have a relatively low sense of feeling at home in the country but at the same time care about being Dutch. In two ways this research tried to provide clear empirical evidence for this expectation.

First, in assessing the robustness of the associations found for autochthony it was examined whether the statistical effects of autochthony was similar for different age groups, boys and girls, level of ethnic identity, perceived multicultural education, and classroom ethnic composition. The presence of similar effects demonstrates that the role of autochthony for attitudes toward immigrants does not depend on these individual and classroom differences and thereby would underscore the general and robust importance of the construct of autochthony beliefs for children’s immigrant attitudes.

Second, given the increased importance of replication in research (e.g., Pashler and Wagenmakers 2012), it is important to ensure that the results could be replicated with different samples of ethnic majority group children and somewhat different measures and operationalizations of group attitudes. The expectations were tested in three separate analyses by using data from three different studies^1^ on children’s attitudes towards school and their academic engagement. In these studies questions on autochthony beliefs and attitudes toward the two most prominent and numerically largest immigrant-origin groups in the Netherlands (of Turkish and Moroccan origin, both around 400,000 people) were included. These two groups have a history of labor migration starting in the late 1960s, followed by a process of family reunification in the mid-1970s which means that nowadays majority group children are mostly exposed to second and third generation immigrants.

## Study 1

### Method

#### Participants and procedure

Participants were 345 children (*M*_*age=*_10.73, *SD=* 0.98; 49.7% girls) who had two ethnic Dutch parents and who self-identified as ethnic Dutch. These children were from 23 classes (grades 4–6: with on average 66.87% Dutch students, *SD=* 25.74, and 5.98% students with at least one parent born in Turkey or Morocco, *SD* = 10.65) in 8 schools in different parts of the country. Participation in the study was voluntary and anonymous and all children with parental permission participated. Together with their classmates, the children completed a questionnaire in their classroom under supervision of their teacher and a research assistant. Apart from the study variables, the questionnaire contained items on children’s relations with their peers and teachers^2^, and experimental vignettes related to diversity. Originally, the sample consisted of 347 ethnic Dutch students, but two cases that had missing scores on the dependent variables were deleted. Overall, very few scores on the items were missing (maximum 1.2% per item) and Little’s MCAR test indicated that missingness was completely at random, *χ*^2^(53) = 63.90, *p=* 0.145.

#### Measures

***Autochthony*** was measured with two items adapted from previous research (Martinovic and Verkuyten 2013): ‘The Netherlands belong to those who came here first’ and ‘Dutch natives can decide what happens in their country’. The response scale ranged from 1 (*No!*) to 5 (*Yes!*) and there was a medium-sized correlation between the items, *r* = 0.32.

***Ethnic identification*** was assessed with three items that have been successfully used in previous research (e.g., Sierksma et al. 2014). On a five-point scale ranging from 1 (No!) to 5 (Yes!) children indicated whether they liked being Dutch, and whether they were proud to be Dutch, and whether they found it important to be Dutch (α = 0.57). Because the autochthony and identification measures had moderate internal reliabilities latent variables were used in the main analyses in order to correct for measurement error (see below).

***Immigrant group attitudes*** was assessed by using the ‘seven faces’ scale developed by Yee and Brown (1992) which ranges from a big smile (coded as 7) to a big frown (coded as 1). Children used this scale to indicate their evaluations of, respectively, Dutch (in-group), and people of Turkish and Moroccan background. All scales were presented on the same page in the questionnaire. The evaluations of Turks and Moroccans were strongly correlated, *r=* 0.81, and therefore averaged into a single measure for out-group attitude. Children’s in-group attitude (*M=* 6.80, *SD=* 0.58) was substantially more positive than their out-group attitude (*M=* 4.49, *SD=* 1.68), *t* (344) = 25.04, *p<* 0.001.

## Results

To examine the distinction between autochthony and ethnic identification, and their unique relations with children’s ethnic attitudes, structural equation modeling (SEM) in Mplus 7 was conducted. As there was no significant classroom variance in the dependent variables (Intraclass Correlations < .06), and to retain an acceptable cases-to-free-parameters ratio (see Kline 2005), we did not include the classroom level in the analyses or examine the role of classroom ethnic composition. Four fit indexes were used: the comparative fit index (CFI), the Tucker Lewis index (TLI), the root mean square error of approximation (RMSEA), and the standardized root mean residual (SRMR). Model fit is considered good if CFI and TLI have values of 0.95 or higher, and RMSEA and SRMR are lower than 0.05. CFI and TLI values larger than 0.9 and RMSEA and SRMR values smaller than 0.1 are considered acceptable (Kline 2005).

First, a confirmatory factor analysis (CFA) was performed to examine whether the autochthony and identification items loaded on two different but correlated factors. Results showed that the fit of the two-factor model was excellent, *χ*^2^ (4) = 3.03, CFI = 1.00, TLI = 1.01, RMSEA = 0.00, SRMR = 0.02, and that the two factors were uncorrelated, *r=* 0.14, *p=* 0.15.^3^

Moreover, the two-factor model fitted the data significantly better than a model that included a single factor for both autochthony and ethnic identification, *χ*^2^_dif_ (1) = 35.78, *p<* 0.001. These findings demonstrate that for the majority group children, autochthony beliefs and ethnic identification were separate constructs.

Next, a Stuctural Equation Model was specified in which children’s in-group attitude and attitude toward immigrants were regressed on the latent factors for autochthony and ethnic identification. MLR as an estimator was used as the distribution of the in-group attitude was non-normal (skewness −4.62, *SE=* 0.13; kurtosis = 32.54, *SE=* 0.26; with 84.9% of the children gave the highest evaluation possible), and one factor for children’s attitude toward the Turkish and Moroccan out-groups was specified. Age and gender were controlled for. The fit of the SEM model was satisfactory, *χ*^2^ (16) = 23.31, CFI = 0.97, TLI = 0.93, RMSEA = 0.036, SRMR = 0.031. Results are shown in Table 1. As expected and indicated by the standardized effects, autochthony had a moderately strong negative relation with children’s immigrant attitude but was unrelated to their in-group attitudes. Conversely, children’s ethnic identification was positively related to their in-group attitudes but it was unrelated to their attitude toward the immigrant-origin groups.

**Table 1:** Regression model for the prediction of group attitudes in study 1

	In-group Attitude	Immigrants Attitude
Autochthony	−0.06	−0.34**
Ethnic identification	0.38**	0.08
Age	−0.01	0.19**
Boys (vs girls)	0.08	0.03
R^2^	0.15	0.14*

Coefficients indicate standardized effects

*p < 0.05, **p < 0.01

To examine the robustness of the effects of autochthony on children’s out-group attitude, a SEM model was specified in which its interactions with identification, age, and gender was added. None of the interactions were significant (*p>* 0.66) which means that the link between autochthony and immigrant attitude was similar for lower and higher ethnic identifiers, younger and older children, and boys and girls.

Lastly, because of the skewed distribution of the in-group attitude measure, an additional analysis was conducted in which this measure was dichotomized and treated as a categorical variable. As in Table 1, there was a positive effect of ethnic ingroup identification but no effects of the other variables.

## Study 2

The first study demonstrated that stronger autochthony belief is related to more negative attitudes toward immigrant-origin groups. The association found was relatively strong and robust across age, gender, and ethnic identification. There were four reasons for conducting Study 2. First, to ensure that the results could be replicated with another sample of majority group children. Second, it could be argued that using two items to measure autochthony in Study 1 is limited and also that these items do not explicitly make the connection between being there first and having the right to decide. Therefore, in Study 2 a third item to the autochthony measure was added. Third, we wanted to examine whether the results are robust or generalize to a different measure of group attitudes and a different immigrant group. Therefore, in Study 2 a measure of trait evaluations was used and the participating children were asked not only about their attitudes toward the main immigrant-origin groups but also toward refugees. Youth tend to have more positive attitudes towards refugees because of feelings of sympathy and pity (e.g., Murray and Marx 2013). However, the importance of autochthony beliefs for out-group attitudes might be similar for both types of out-groups. Fourth, perceived multicultural education as a meaningful factor for children’s immigrant attitudes was considered and the role of classroom ethnic composition was examined as there was significant variation between classrooms (see below). Multicultural education tries to foster equality and inclusion which tends to improve intergroup attitudes (Schachner et al. 2016; Verkuyten and Thijs 2013). This could mean that the associations found are due to perceived multicultural education as a third confounding factor. Likewise, native majority students in more diverse, less segregated classrooms may be more open to ethnic diversity because they have more opportunities for intergroup contact (Thijs and Verkuyten 2014). Therefore, it was examined whether autochthony predicts immigrant attitudes independently of perceived multicultural education and classroom ethnic composition.

### Method

#### Participants and procedure

One-hundred-and-ninety-five native Dutch students (54.5% girls) from 22 classes (grades 4–6) in 15 schools in different parts of the Netherlands participated in this research. Together with their classmates, they took part in a larger short-term longitudinal study on teachers’ classroom dealings with ethnic diversity. That larger study consisted of three waves and at all waves children anonymously filled in questionnaires in their classroom under supervision of their teacher or a research assistant. Apart from questions related to ethnic diversity, the questionnaires included items on children’s experiences with their teachers and their peer relations.

Again, children participated voluntarily and there was consent from their parents and the possibility to opt out. In the present study data from Wave 3 (June and July) contained questions on autochthony and were therefore used. However, the Wave 1 data (October and November) were relied on to select the participants because only these data included information about the ethnicity of the parents. At Wave 1, i.e. seven to nine months prior to completing the autochthony and group attitude measures, the children were on average 10.13 years old (*SD=* 0.82).

To be included in the current analysis, the children had to self-identify as ethnic Dutch and to indicate that both of their parents were of Dutch origin. Originally, 212 participants were selected in this manner. Yet as few scores on the variables were missing (<5%), and the pattern of missing values appeared to be completely at random, *χ*^2^(52) = 56.947, *p=* 0.30, listwise deletion was used.

### Measures

***Autochthony*** was measured with the same two items as in Study 1 plus one additional item, namely “The people who came to live here [the Netherlands] first may decide what might change”. The items had a response scale ranging from 1 (*No!*) to 5 (*Yes!*) and yielded a Cronbach’s α of 0.82.

***Ethnic identification*** was measured in this dataset with two of the identification questions used in Study 1. Children were asked whether they liked being Dutch and whether they were proud to be Dutch, using response scales from 1 (*Not all*) to 5 (*Very much*). The correlation between both items was 0.54.

***Perceived multicultural education*** was assessed with three items taken from previous research in the Netherlands (see Verkuyten and Thijs 2013). Children were asked ‘Does your teacher ever say that all cultures should be respected?’, ‘Does your teacher ever say that it is wrong to discriminate?’, and ‘Does your teacher ever say that people from all cultures are equal?’. The response scale ranged from 1 (*Absolutely never*) to 5 (*Very often*) with α = 0.83.

***Classroom ethnic composition*** was operationalized as the proportion of Dutch children in each class (*M*_*%Dutch=*_52.25, *SD=* 27.22). This proportion was strongly related to the proportion of students who self-identified as Turkish or Moroccan (*r=* −0.77) but considerably less skewed (*M*_*%Turkish/Moroccan*_*=* 16.81, *SD=* 24.84). Confirmatory factor analysis in Mplus with ML as the estimator showed that the items for the independent variables (autochthony, ethnic identification, and perceived multicultural education) loaded on three corresponding factors without cross-loadings or error correlations, χ^2^ (17) = 30.007, CFI = 0.978, TLI = 0.964, RMSEA = 0.060, SRMR = 0.044.

***Immigrant attitude*** was assessed through trait evaluations of Moroccan and Turkish peers (see Brown and Bigler 2002) that have been successfully used in previous research in the Netherlands (e.g., Thijs 2017). More specifically, participants indicated whether they thought that most of the children in each group were, “honest”, “fun to play with”, and “eager to help you”. The same traits were used to measure the in-group attitude. The response scale ranged from 1 (NO, certainly not!) to 5 (YES, certainly!). For each of the three groups the evaluations yielded a reliable scale: Cronbach’s alpha was 0.79 for the evaluation of the Dutch in-group, and respectively, 0.89 and 0.88 for the Moroccan and Turkish out-groups. The attitudes toward the two out-groups were strongly correlated (*r=* 0.71). Therefore, and in keeping with Study 1, these two measures were averaged in one measure for immigrant attitude.

***Attitude toward refugees*** was measured with two items: “Some people think that there are too many refugees coming to the Netherlands and others don’t. What do you think?” with a response scale ranging from 1 (Absolutely not too many!) to 5 (Far too many!), and “Some people think that refugees are helped too little and other people think they are helped too much. What do you think?” with a response scale from 1 (*Far too little!*) to 5 (*Far too much!*). The correlation between these items was 0.60. Items were recoded so that higher scores indicated a more positive attitude.

Confirmatory factor analysis in Mplus showed that the items for the dependent variables loaded on different factors (without cross-loadings or error correlations) for the attitudes toward, respectively, the Dutch in-group, Moroccans, Turks, and refugees, χ^2^ (38) = 89.907, CFI = 0.956, TLI = 0.936, RMSEA = 0.080, SRMR = 0.030. Moreover, there was negligible drop in model fit when a higher-order factor was specified for the attitude toward the two immigrant-origin groups, χ^2^_dif_(1) = 0.026, *p=* 0.87, which justifies the decision to examine children’s attitudes toward Moroccans and Turks as a single out-group.

### Results

The intercorrelations and means of the main variables are shown in Table 2. As in Study 1, children reported a more positive evaluation of their in-group compared to the immigrant out-groups, *t* (194) = 10.91, *p<* 0.001. Compared to Study 1 the distribution of children’s in-group evaluation was considerably more normal (skewness = −0.26, *SE* = 0.17, kurtosis = −0.91, *SE=* 0.35). To examine the unique contribution of children’s autochthony beliefs on their attitudes, a multivariate regression model in Mplus was specified. Given sample size restrictions, observed variables were analyzed. Moreover, the multilevel structure was taken into account, as children’s attitudes toward refugees differed systematically between classrooms (Intraclass correlation = 0.18). Because of the limited number of classrooms children’s attitudes toward respectively immigrants and refugees and their in-group were analyzed in separate models. Whereas ML was used as the estimator for the first model, MLF was used for the in-group model due to estimation problems. In both models these attitudes were regressed on autochthony, ethnic identification, and perceived multicultural education on the individual level, and the proportion of Dutch students on the classroom level. Prior to the analyses all continuous variables were standardized (*z* scores), and age and gender were controlled for. Results are given in Table 3.

**Table 2:** Intercorrelations, means, and standard-deviations for main variables in study 2

	1	2	3	4	5	6	*M (SD)*
1. Autochthony							2.84 (1.12)
2. Ethnic identification	0.20**						4.25 (0.73)
3. Perceived multicultural education	−0.08	0.13					2.85 (1.02)
4. % Dutch students^a^	0.07	−0.06	−0.47*				52.25 (27.22)
5. In-group attitude	0.27**	0.30**	0.07	0.04			4.24 (0.61)
6. Immigrants attitude	−0.28**	−0.09	0.15*	0.09	0.23**		3.53 (0.82)
7. Refugee attitude	−0.40**	−0.16*	0.02	0.54**	0.05	0.48**	2.85 (1.00)

^a^Intercorrelations and mean and standard-deviation of this variable were calculated at the class level

*p < 0.05; **p < 0.01

**Table 3:** Regression model for the prediction of group attitudes in study 2

	In-group Attitude	Immigrants Attitude	Refugee Attitude
*Level 1 variables*			
Autochthony	0.23**	−0.25**	−0.33**
Ethnic identification	0.25**	−0.07	−0.05
Perceived multicultural education	0.07	0.11	0.06
Age	−0.05	0.13	−0.05
Boys (vs girls)	0.04	0.20	−0.08
*Level 2 variable*			
% Dutch students	0.05	−0.00	0.19*
*Residual variance*			
Level 1 (within classrooms)	0.854	0.864	0.712
Level 2 (between classrooms)	0.001	0.003	0.091

MLF for in-group attitude

*p< 0.05, **p< 0.01

Autochthony had a positive effect on the attitude toward the in-group, and moderate and large negative effects on children’s attitudes toward, respectively, immigrant-origin children and refugees. Further inspection showed that, although the effect of autochthony was larger for refugees versus immigrants, the difference between the effects was not significant, χ^2^_dif_(1) = 1.553, *p=* 0.22. Next and similar to Study 1, children’s ethnic identification was related only to their in-group attitude. Perceived multicultural education was positively associated with out-group attitudes, and, somewhat surprisingly, the proportion of Dutch students had a positive effect on children’s evaluations of refugees.

In an additional set of analyses it was tested whether autochthony interacted with ethnic identification, perceived multicultural education, classroom ethnic composition, age, and gender, and to avoid model under-identification, separate models for each dependent variable were estimated. None of these two-way interactions were significant, *p*_*s*_*>* 0.15, indicating the robustness of the effects of autochthony on the attitudes towards immigrants and refugees.

Thus the findings in Study 2 are consistent with those in Study 1 and demonstrate that the importance of autochthony beliefs for immigrant attitudes is independent of ethnic identification, perceived multicultural education, and classroom ethnic composition, and generalizes to another sample of majority group children, another group attitude measure, and another type of migrant group (refugees).

## Study 3

The aim of Study 3 was to investigate whether feeling at home in the Netherlands as a country is a relevant condition for the association between autochthony and immigrant attitudes. It was expected that for majority group children with relatively low home feeling, autochthony is more strongly negatively associated with immigrant attitudes. The reason is that children are more likely to adopt a ‘primordial reasoning’ when their perceived ownership right to feel at home in their own country is undermined *(*Duyvendak 2011). Furthermore, this is especially likely for children who at the same time consider their Dutch identity an important aspect of their sense of self. According to social identity theory (Tajfel and Turner 1979), relatively low identifiers should be less concerned about their in-group and less inclined to justify negative out-group attitudes. Thus a three-way interaction between autochthony, home feeling and ethnic identification on attitudes toward immigrant was expected.

### Method

#### Participants and procedure

The sample consisted of 337 ethnic Dutch students from 36 classes (grades 4–6; *M*_*%Dutch*_*=* 41.48, *SD=* 26.98) in 16 schools in different parts of the Netherlands. These children (*M*_*age*_ = 10.84 years, *SD* = 0.94; 52.8% girls) took part in a short-term longitudinal study on classroom dealings with ethnic diversity. The study consisted of two waves. Wave 1 was halfway through the school year (January-March) and Wave 2 was at the end of the school year (June and July). During both waves children were surveyed under similar conditions. They completed a questionnaire in their classroom, together with their classmates, and under supervision of their teacher and/or a research assistant. As in Study 2, the questionnaires included questions related to ethnic diversity and children’s experiences with their teachers and peers. Participation in the study was voluntary and anonymous and all children with parental consent participated.^4^ The children were selected based on their ethnic self-labeling at Wave 2 but the additional criteria were used that this self-labeling should be similar to that of Wave 1 and that both parents of the children should be born in the Netherlands. Originally 359 children could be selected in this way but because there were few missing values on the variables (≤2%) and missings appeared to be at random, χ^2^(40) = 39.506, *p=* 0.49, listwise deletion was used.

#### Measures

All measures were collected during Wave 2, except children’s perception of multicultural education which was assessed at Wave 1.

***Autochthony*** was measured with the same three items and response scales used in Study 2 (α = 0.71).

***Ethnic identification*** was measured with the same three items as in Study 1 (α = 0.66).

***Home feeling*** was assessed with three items that have been successfully used in previous research among children in the Netherlands (Verkuyten et al. 2014). On a five-point scale ranging from 1 (*No!*) to 5 (*Yes!*) children indicated whether they felt at home in the Netherlands, whether they were proud of the Netherlands, and whether they liked it in the Netherlands (α = 0.86).

***Perceived multicultural education*** was measured with the same three items that were used in Study 2 (α = 0.74).

Confirmatory factor analysis in Mplus (on *n=* 359, and with ML as the estimator) showed that these four measures corresponded to four different factors with independent error terms and no cross-loadings, χ^2^ (48) = 146.738, CFI = 0.930, TLI = 0.903, RMSEA = 0.076, SRMR = 0.057. The fit of this model was substantially better than that of a three-factor model in which ethnic identification and feeling at home loaded on one factor, χ^2^_dif_(3) = 104.601, *p* < 0.001. Further, because the items for home feeling had a skewed distribution, CFA was also conducted with MLR as an estimator. Model fit was acceptable after allowing an error correlation between the first and the last item for home feeling, χ^2^ (47) = 107.602, CFI = 0.946, TLI = 0.924, RMSEA = 0.060, SRMR = 0.053.

***Attitude towards immigrants*** was again measured with the ‘seven faces’ scale (Yee and Brown 1992) in relation to people of Turkish and Moroccan background. As these evaluations were strongly related (*r=* 0.77) these measures were combined into a single out-group attitude score. In this study the attitude toward refugees was not assessed and in-group attitude was measured with the same ‘seven-faces’ scale.

### Results

The intercorrelations and means of the main study variables are shown in Table 4. As in the previous two studies, children reported a more positive attitude toward their in-group than toward the immigrant out-groups, *t* (336) = 23.07, *p<* 0.001. As in Study 1, the distribution of the in-group attitude was non-normal (skewness −3.99, *SE=* 0.13; kurtosis = 21.55, *SE=* 0.27). To examine the unique contribution of children’s autochthony beliefs on their attitudes a series of multivariate regression models in Mplus was specified. To account for the non-normal distributions of in-group attitude MLR was used as an estimator. Moreover, given sample size restrictions, observed variables were analyzed and the multilevel structure was taken into account as there were substantial differences between classrooms in children’s evaluations of the immigrant groups (Interclass correlation = 0.17). Prior to the analyses, all continuous variables were standardized (*z* scores) at the individual level.

**Table 4:** Intercorrelations, means, and standard-deviations for main variables in study 3

	1	2	3	4	6	7	*M (SD)*
1. Autochthony							2.77 (1.02)
2. Ethnic identification	0.23**						4.04 (0.75)
3. Feeling at home	0.13*	0.50**					4.57 (0.61)
4. Perceived multicultural education	−0.08	−0.07	0.06				3.12 (0.92)
5. % Dutch students	−0.03	0.15	0.04	−0.52**			41.48 (26.98)
6. In-group attitude	0.15**	0.30**	0.38**	−0.00	−0.06		6.73 (0.71)
7. Immigrants attitude	−0.33**	−0.11*	0.08	0.24**	0.08	−0.17	4.38 (1.79)

*p < 0.05; **p < 0.01

In the first model, children’s in-group attitude and immigrant attitude were regressed on age, gender, and the classroom proportion of Dutch students, as well as on autochthony, ethnic identification, home feeling, and perceived multicultural education. The results of the model are shown in Table 5 (Model 1). Autochthony had a small positive effect on the evaluation of the in-group and again a medium-sized negative effect on the evaluation of the immigrant-origin groups. These effects were independent of ethnic identification which was positively related to the evaluation of the in-group, and in this study also negatively to the evaluation of the immigrant out-groups. Home feeling was positively related to both in-group attitude and the attitude toward immigrants. Additionally, boys were more positive about the majority in-group, and similar to Study 2, perceived multicultural education was positively related to children’s attitude toward immigrants. There were no effects of the proportion of Dutch students.

**Table 5:** Regression model for the prediction of group attitudes in study 3

	Model 1		Model 2	
	Majority Attitude	Immigrants Attitude	Majority Attitude	Immigrants Attitude
*Level 1 variables*				
Autochthony (AUT)	0.10**	−0.32**	0.12**	−0.39**
Ethnic identification (EI)	0.13**	−0.10*	0.13**	−0.14**
Feeling at home (FH)	0.30**	0.15**	0.24**	0.12**
AUT * EI			−0.02	−0.01
AUT * FH			−0.06	0.11*
EI * FH			−0.09	0.02
AUT * EI * FH			−0.05	0.15**
Multicultural education	−0.01	0.15*	−0.01	0.14
Age	0.01	−0.01	0.01	−0.03
Boys (vs girls)	0.25*	−0.03	0.25*	−0.03
*Level 2 variable*				
% Dutch students	0.03	−0.12	0.02	−0.13
*Residual variance*				
Within classrooms	0.812	0.708	0.798	0.688
Between classrooms	0.005	0.100	0.004	0.098

*p< 0.05, **p< 0.01

**Fig. 1:**
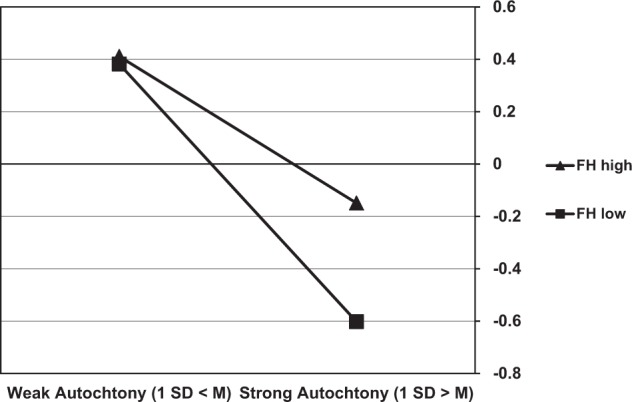
Effects of autochthony on the attitude towards immigrants (standardized) for low (1 SD > M) versus high (1 SD > M) levels of home feeling (FH)

In the second model, the two- and three-way interactions between autochthony, ethnic identification and home feeling were added. As shown in Model 2 (in Table 5) none of the interactions were significant for the attitude toward the ethnic in-group. However, for the attitude toward immigrants the two-way interaction between autochthony and home feeling, and the three-way interaction between autochthony, ethnic identification and home feeling were both significant. Simple slope analyses were conducted to decompose these interactions. The two-way interaction between home feeling and autochthony was first examined by calculating the effect of autochthony at low (1 SD > M) versus high (1 SD > M) versus levels of home feeling. As shown in Fig. 1, this effect was stronger in the former case, *b=* −0.49, *se* = 0.07, versus *b=* −0.28, *se* = 0.08, *p*_s_ < 0.001. Next, it was investigated whether this two-way interaction was different for children with relatively high (1 SD > M) versus low (1 SD > M) levels of ethnic identification. This showed that the two-way interaction was positive and significant for the high identifiers, *b* = 0.26, *se* = 0.07, *p<* 0.001, but not for the low identifiers, *b* = −0.04, *se* = 0.06, *p=* 0.45.

**Fig. 2:**
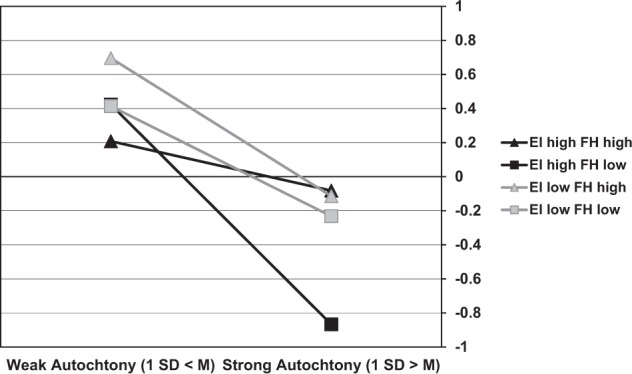
Effects of autochthony on attitude towards immigrants (standardized) depending on ethnic identification (EI) and feeling at home (FH), with ‘low’ and ‘high’ denoting, respectively one standard deviation below and above the means

Figure 2 shows the effects of autochthony on children’s immigrants attitude for different levels of ethnic identification and feeling at home. The effect of autochthony on immigrants attitude was strongest at high levels of ethnic identification combined with relatively low levels of home feeling, *b=* −0.66, *se* = 0.12, *p<* 0.001, and weakest at high levels of ethnic identification combined with high levels of home feeling, *b=* −0.14, *se* = 0.09, *p=* 0.12. Moreover, the attitude was clearly most negative among children who endorsed autochthony, did not feel at home in the Netherlands and considered their Dutch identity an important part of their sense of self.

Two further models (not in Table 5) examined whether autochthony interacted with the remaining predictors (respectively, with perceived multicultural education and the proportion of Dutch students in Model 3a, and with age and gender in Model 3b) by adding these two-way interactions to the previous model (Model 2). None of these two-way interactions were significant (*p*_*s*_*>* 0.10).

Finally, in light of its skewed distribution, a logistic regression was performed on dichotomized version of the in-group attitude variable (just as in Study 1). This analysis failed to replicate the positive main effect of autochthony (Table 5), OR 1.373, *p=* 0.29 which means that positive main effect should be interpreted with care.

The findings of Study 3, again, show that autochthony is negatively associated with attitudes toward immigrant-origin groups. However, this association is stronger for those children who have a lower feeling of being at home while at the same time self-identify as Dutch. This suggests that autochthony functions as a justifying belief for native children who care about being Dutch but do not feel at home in the Netherlands.

## Discussion

The notion of ownership is widespread, pervasive, and applied to a range of objects including places in children’s lives at home, in school, their neighborhood and the country they live in (e.g., Barrett 2007). Furthermore, the belief that a territory belongs to those who arrived first is often self-evidently used to claim rights (e.g., “first nations, indigenous people”) and to exclude outsiders and newcomers (Ceuppens 2011; Geschiere 2009). Those who were somewhere first are typically considered to own the place with the related rights of usage and “gatekeeping” which provide justified reasons to exclude others (Merrill 1998). Yet, research on children’s intergroup attitudes has not paid systematic attention to these issues and the current research is the first one that has examined the importance of autochthony beliefs for children’s attitudes toward immigrant groups.

In three studies, it was found that autochthony belief and ethnic identification were empirically distinct constructs, and that autochthony was not strongly and consistently associated with in-group attitude. These findings suggest that first arrival is a basis for inferring ownership and not so much for a sense of group belonging or in-group liking (Verkuyten et al. 2014). Furthermore, the findings demonstrate that ethnic majority group children with stronger autochthony beliefs had more negative attitudes toward immigrant-origin groups and refugees which suggests that both groups of newcomers were perceived in similar ways. This expected association was found among three different samples, by using different measures of ethnic attitudes, and by controlling for ethnic identification, perceived multicultural education and classroom composition. Furthermore, the importance of autochthony belief for out-group attitudes was robust across gender, age, level of ethnic identification, and level of perceived multicultural education. Thus there was clear and substantial evidence (small to medium effect sizes) that majority children who believe that their in-group was here first and therefore owns the country, have more negative attitudes toward immigrant groups.

Additionally, Study 3 showed that this association was especially strong among children who felt less at home in their country. This finding is reminiscent of the widespread discourse in many Western countries that argues that immigration makes majority members feel like foreigners in their own country (Duyvendak 2011). Although they were here first and therefore have the right to feel at home in their own country (see quote heading this paper), they do not feel at home anymore and therefore should take back control, the argument goes. This discourse is particularly appealing to people who identify with the majority group (Martinovic and Verkuyten 2013). Children are aware of the societal debate on immigration and anti-immigration sentiments (Brown 2011) and similar to the prevailing discourse, it was found that especially for children with high ethnic identification, the combination of autochthony beliefs with not feeling at home was associated with negative attitudes. Interestingly, in the three studies the importance of autochthony beliefs for the attitudes did not depend on the level of ethnic identification. This indicates that autochthony belief is not a more relevant justifying belief for higher compared to lower identifiers. However, when at the same time there is a sense of not feeling at home, the level of identification seems to matter. This finding can be interpreted in terms of social identity development theory (Nesdale 2008) which proposes that ethnic prejudice appears when children who identify relatively strongly with their group feel that their in-group position or well-being is undermined in some way by members of ethnic out-groups.

As with all studies, there are limitations to our research that provide possible directions for future research. First, although the findings are consistent and robust across the three studies, future research could try to use more extensive measures and try to assess additional constructs (e.g., feelings of outgroup threat, moral reasoning) which could increase the explained variance. For example, the measure of country belonging did not ask whether children felt less at home in their country because of the arrival and presence of immigrant groups. Considering the societal discourse (Duyvendak 2011) and research findings (Huijnk and Andriessen 2016) this is very likely and the pattern of findings is in agreement with this interpretation, but future research could assess this directly. Future research could also examine the associations longitudinally or using an experimental design. There is experimental evidence that children consider first arrival a legitimate reason for claiming personal as well as collective territorial ownership (Verkuyten et al. 2015a, 2015b). Yet, in contrast to research among adults (Martinovic et al. 2019), there is among children no experimental evidence that collective ownership based on primo-occupancy influences out-group attitudes.

Second, the role of autochthony beliefs for attitudes toward immigrant groups in the Netherlands was examined. Similar to most European countries, in the Netherlands there is a large native majority population. This is different from immigration countries such as the United States, Canada and Australia that were inhabited first by indigenous groups of Native Americans and Aborigines. This could mean that for the White majority population in these countries collective ownership based on autochthony beliefs is less useful for justifying anti-immigrant attitudes. However, a sense of ownership with the related entitlements might also be derived from being *earlier* than later immigrants and from having invested in and developed the land (Verkuyten and Martinovic 2017). First arrival might be disregarded when later arrivers think that they are the ones who have made the land prosper. There will be many situations in which place ownership inferences are not based on the first arrival assumption alone, or at all. A lay belief can be interpreted and used in more than one way across situations: when one of its interpretations is not useful or appropriate in a particular context, another interpretation can be invoked (Levy et al. 2008). Future research could examine these possibilities in different national contexts, including in contexts of territorial disputes, such as in the Middle East (Zebian and Rochat 2012).

Third, autochthony belief was examined in relation to the country but there are many other places in relation to which youth makes first arrival ownership claims, such as when children convert a place in their home or outside in their private playing area (Factor 2004), and in gang behavior among youth (Childress 2004; Kintrea et al. 2008). Having a sense of collective ownership (“ours”) can involve many different settings with similar exclusionary consequences. Hence, it could be examined whether the present findings also apply to neighborhoods, schools, play areas and other places in which children feels that they own the place because they were there first. It is likely that a sense of collective ownership based on primo-occupancy is a more general process that has implications for children’s prejudices and forms of social exclusion in a range of settings.

Fourth, concern with ownership is already evident in young children but the evidence relates primarily to ownership of objects and ideas and therefore it is unclear how young children reason about place ownership and how this develops. We focused on older children and future studies could use a longitudinal design to examine, for example, at what age children develop an understanding that land, or a particular place, can be owned and the type of information that they use to infer place ownership and the related entitlements. With such a design it can also be examined how autochthony beliefs develop and whether and how these beliefs, for example, relate to the ethnic and national identity development of youth. Furthermore, such a design would be useful for examining where children’s autochthony beliefs come from and the roles that parents, teachers and peers play in developing these beliefs and using them to justify negative attitudes towards immigrant groups.

## Conclusion

This research has tried to make a novel and first contribution to the intergroup developmental literature (Rutland et al. 2010) by focusing on the importance of autochthony beliefs. This literature has examined out-group attitudes in relation to, for example, identification processes, threats, group norms and moral reasoning, and there is work on lay theories and justifying beliefs. Extending this literature to the important field of perceived ownership (Nancekivell et al. 2013), we showed that being here first is a relevant consideration for children’s out-group attitudes. This corresponds to research among adults that has demonstrated that notions of autochthony are central in “sons of soil” conflicts (Côté and Mitchell 2017) and that people use these notions in territorial disputes and in exclusionary behavior and negative feelings towards outsiders and immigrants (Ceuppens and Geschiere 2005; Geschiere 2009). For children, being here first can be an acceptable reason for claiming collective ownership of a place and this claim can have negative consequences for newcomers. The reception and accommodation of immigrants and refugees is one of the major social challenges of our times. Understanding how majority group children think about these newcomers is important for trying to address their feelings and concerns. Hopefully, the current research adds to a further understanding of children’s out-group attitudes and thereby helps to evaluate and further think about initiatives to improve their attitudes.

## Footnotes

1 The different studies involved three independent data collections. Two of the schools participated in Study 2 and Study 3. However, there was a two-year difference between both data collections, and for one of the schools the children were in respectively in grade 6 and grade 8 in the earlier and the later data collections.

2 In a previous research (Masked Reference), the immigrant attitude measures analyzed in the current Studies 1 and 3 and those from a prior wave of Study 2 were predicted from the quality of the student-teacher relationship. Likewise, two of these studies controlled for perceived peer acceptance. Student-teacher relationship quality and perceived peer acceptance were not included in the present studies as they were unrelated to autochthony. It was not theoretically relevant to include other variables from the datasets in the present analyses.

3 Standardized factor loadings were, respectively, 0.86 and 0.38 for the two autochthony items, and 0.66, 0.80, and 0.47 for the three identification items.

4 There was little attrition. Of the total sample (including participants and their classmates), 94.8% participated at Wave 1 and 93.6% at Wave 2.
